# European Code Against Cancer, 5th edition – hormone replacement therapy, other common medical therapies and cancer

**DOI:** 10.1002/1878-0261.70158

**Published:** 2025-11-18

**Authors:** Mangesh A. Thorat, Marc Arbyn, David Baldwin, Xavier Castells, Solveig Hofvind, Urska Ivanus, Carlo Senore, Esther Toes‐Zoutendijk, Carlijn van der Aalst, Carlos Canelo‐Aybar, Fiorella Karina Fernández‐Sáenz, Ariadna Feliu, Hajo Zeeb, Andre L. Carvalho, Erica D'Souza, David Ritchie, Carolina Espina, Iris Lansdorp‐Vogelaar, Andrea DeCensi

**Affiliations:** ^1^ Centre for Cancer Screening, Prevention and Early Diagnosis, Wolfson Institute of Population Health Queen Mary University of London UK; ^2^ Breast Services Homerton University Hospital London UK; ^3^ Unit of Cancer Epidemiology, Belgian Cancer Centre Sciensano Brussels Belgium; ^4^ Department of Respiratory Medicine Nottingham University Hospitals NHS Trust UK; ^5^ Population Health University of Nottingham UK; ^6^ Department of Epidemiology and Evaluation, Hospital del Mar Medical Research Institute (IMIM) Pompeu Fabra University Barcelona Spain; ^7^ Department of Breast Cancer Screening, Cancer Registry Norwegian Institute of Public Health Oslo Norway; ^8^ Cancer screening and clinical genetic division Institute of Oncology Ljubljana Slovenia; ^9^ Epidemiology and screening unit – CPO University hospital Città della Salute e della Scienza Turin Italy; ^10^ Department of Public Health Erasmus MC – University Medical Centre Rotterdam The Netherlands; ^11^ Institut de Recerca Sant Pau (IR SANT PAU) Barcelona Spain; ^12^ Centro Cochrane Iberoamericano Barcelona Spain; ^13^ Environmental and Lifestyle Epidemiology Branch International Agency for Research on Cancer Lyon France; ^14^ Department of Primary Care and Public Health, School of Public Health Imperial College London UK; ^15^ Department of Prevention and Evaluation Leibniz – Institute for Prevention Research and Epidemiology ‐ BIPS GmbH Bremen Germany; ^16^ Early Detection, Prevention, and Infections Branch International Agency for Research on Cancer Lyon France; ^17^ Division of Medical Oncology, Department of Medicine Ente Ospedaliero Ospedali Galliera Genoa Italy; ^18^ Breast Unit Champalimaud Foundation Lisbon Portugal

**Keywords:** aspirin, breast cancer, European Code Against Cancer, hormonal contraceptive, Hormone replacement therapy, menopause

## Abstract

Several medical therapies modify the risk of developing certain cancers in an individual. The aim of this paper was to provide the scientific justification for the 5th edition of the European Code Against Cancer (ECAC5) recommendation on the use of hormone replacement therapy (HRT) and other drugs used at population scale, such as hormonal contraceptives and aspirin. HRT modifies the risk of developing certain cancers in an individual. Except for vaginal oestrogens, all forms of HRT are associated with an increased breast cancer risk; the risk of serous ovarian cancer and endometrial cancer may also be increased. Despite such an increase in cancer risk, HRT often remains the only option for the management of certain menopausal symptoms for the restoration of quality of life and mental health. Therefore, the ECAC5 recommends using HRT for bothersome menopausal symptoms only after a thorough discussion with a healthcare professional and limiting its use for as short a duration as possible. On review of up‐to‐date evidence for hormonal contraceptives and aspirin, the ECAC5 Working Group elected not to make a recommendation for the average‐risk general population regarding the use of these therapies.

## Graphical abstract

The 5th edition of the European Code Against Cancer (ECAC5) contains 14 recommendations on cancer prevention. Here, we update the cancer prevention recommendations regarding the use of hormone replacement therapy (HRT), which is positioned as recommendation number 13 of the ECAC5. Individuals should use HRT for menopausal symptoms that are not alleviated with nonhormonal interventions. The use of HRT in asymptomatic individuals is strongly discouraged. HRT should be started only after a thorough discussion of the benefit‐harm balance with a healthcare professional, and it should be used for the shortest duration possible.

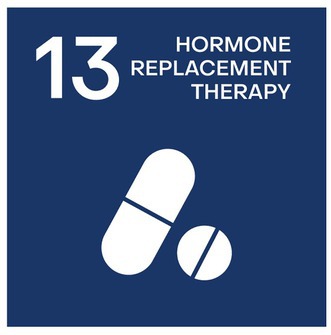

**Abbreviations**
AI: aromatase inhibitors
CBT: cognitive behavioural therapy
CEE: conjugated equine oestrogen
CHD: coronary heart disease
CPRD: Clinical Practice Research Datalink
CRC: colorectal cancer
ECAC: European Code Against Cancer
ECAC4: European Code Against Cancer, 4^th^ edition
ECAC5: European Code Against Cancer, 5^th^ edition
EU: European Union
HR: hazard ratio
HRT: hormone replacement therapy
MBD: Mammographic breast density
MPA: medroxyprogesterone acetate
NCD: non‐communicable diseases
NICE: UK National Institute for Health and Care Excellence
OR: Odds Ratio
PAF: population attributable fraction
PYAR: person‐years‐at‐risk
RR: relative risk
SERM: selective oestrogen receptor modulators
SRs: systematic reviews
USPSTF: US Preventive Services Task Force
WHI: Women's Health Initiative
WHS: Women's Health Study

## 1 Introduction

Several medical interventions modify the risk of developing cancer in an individual. These include hormone replacement therapy (HRT) [1], hormonal contraceptive agents, ionising radiation [2, 3] and antineoplastic agents [4], which increase an individual's risk of developing cancer and are classified as carcinogens by the International Agency for Research on Cancer (IARC/WHO) [5]. Several other pharmacological agents such as aspirin [6, 7, 8], selective oestrogen receptor modulators (SERMs) such as tamoxifen [9, 10], aromatase inhibitors (AIs) such as anastrozole [11, 12] and exemestane [13], and 5‐alpha reductase inhibitors (5‐ARIs) such as finasteride [14, 15] and dutasteride [16] reduce the risk of developing specific cancers particularly in certain high‐risk populations [17, 18]. Treatment of infections such as *Helicobacter pylori* (*H. pylori*) [19, 20], hepatitis B [21, 22] and hepatitis C infection [23, 24] also reduces the risk of developing specific cancers. Medical interventions to test and treat infections of cancer‐causing organisms are reviewed in a separate paper [25]. The diagnostic use of ionising radiation, therapeutic use of antineoplastic agents and therapeutic interventions to prevent cancer in high‐risk populations are outside the scope of this paper.

**Fig. 1:**
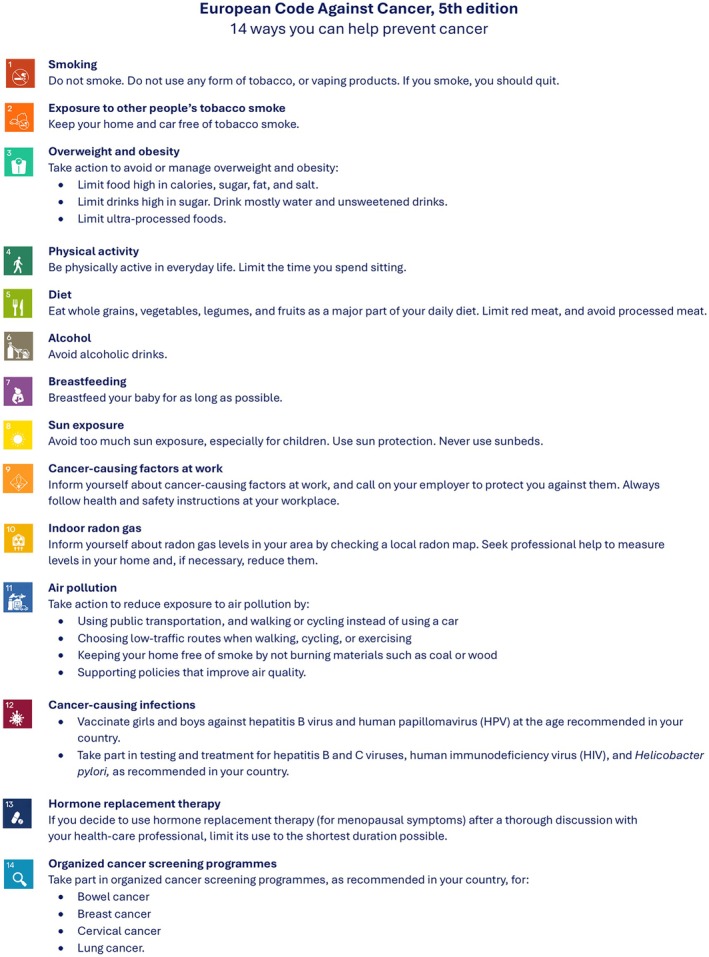
European Code Against Cancer, 5th edition: recommendations for individuals. The 14 recommendations of the European Code Against Cancer, 5th edition (ECAC5) adopted by the Scientific Committee of the ECAC5 project. © 2025 International Agency for Research on Cancer / WHO. Used with permission. [Correction added on 08 January 2026 after first online publication: The legend was edited to match other articles included in the same thematic issue. The credit line was updated as well.]

The European Code Against Cancer (ECAC) is an initiative of the European Commission designed to provide clear, evidence‐based recommendations for cancer prevention, accessible to the general public. The current 5th edition has been coordinated by IARC as part of the World Code Against Cancer Framework, launched by IARC in 2022 to support the development of region‐specific Codes tailored to distinct epidemiological and socio‐economic contexts [26]. The 5th edition of the ECAC (ECAC5) builds on the 4th edition (ECAC4) [27], also coordinated by IARC, by integrating the latest scientific evidence in cancer prevention. For the first time, ECAC5 is aimed not only at individuals (Fig. 1) but also at policymakers, including 14 complementary recommendations on a population level that may reinforce the 14 recommendations for individuals (Supplementary material—Annex S1). A specific methodology has been constructed for use in the update of ECAC5 as described in a separate paper [28]. Further details about the ECAC5 project are provided in a separate paper [29].

In this paper, we review the contemporary evidence regarding common medical therapies in relation to cancer risk in the general population with the main objective of providing the scientific justification for the ECAC5 recommendation on HRT.

## 2 Prevalence and trends of HRT use in the European Union (EU)/Europe

The trends and patterns of HRT use in the 20th century have been reviewed in the previous ECAC4 paper [27]. The Women's Health Initiative (WHI) trial, a double‐blind, placebo‐controlled trial included two components: the oestrogen plus progestin component (combined HRT) in postmenopausal women aged 50–79 years with an intact uterus at baseline and the oestrogen‐alone component in postmenopausal women aged 50–79 years with prior hysterectomy [30]. On 31 May 2002, after a mean of 5.2 years of follow‐up, the data and safety monitoring board recommended stopping the combined HRT trial component because the test statistic for invasive breast cancer exceeded the stopping boundary for this adverse effect and the global index statistic supported risks exceeding benefits [31]. This early stoppage of the trial, primarily due to excess breast cancer risk [32] resulted in a rapid decline in the use of HRT worldwide. Such a rapid decline in HRT use may have contributed to a decline in breast cancer incidence in the United States from mid‐2002 to mid‐2003 [33], although an alternative hypothesis has been proposed [34]. A similar reduction in the use of HRT occurred in other parts of the world and HRT use continued to decline until 2010 [35].

In Europe, the HRT use in women 45–69 years varied considerably between countries ranging from less than 5% to more than 25% in the Year 2002 [35]. However, a substantial decrease in the rate of use ranging from 50% to 77% occurred in all countries between 2002 and 2010 and by the end of 2010, less than 10% of women aged 45–69 in all countries, except in Finland, used HRT [35]. The variations in the use of HRT among different European countries are underpinned by variation in familiarity, attitudes as well as experiences regarding treatment of menopausal symptoms among women from these countries [36]. The decline in the use of HRT however started reversing around 2010. Burkard et al. [37] reported that between 1998 and 2001, the proportion of HRT initiation was around 1.7%, which halved by 2005 (0.8%), and increased again up until 2015. The authors [37] also observed trends towards a greater use of oestrogen‐alone therapy and vaginal HRT, and also a trend of lower HRT dose being used after 2002/2003. Similarly, in a study from the United Kingdom (UK), Alsugeir et al. [38] reported that the incidence rate of prescribing of HRT increased from 5.01 in 2010 to 18.16 per 1000 person‐years‐at‐risk (PYAR) in 2021. This increase was largely driven through the use of transdermal formulations, which increased from 1.48 to 14.55 per 1000 PYAR in 2010 and 2021, respectively [38]. A study from Denmark by Meaidi et al. [39] also showed that the prevalence of Danish women using vaginal oestrogen increased from 8.5% in the Year 2007 to 10.2% in 2013. The use was highest in women aged 60–74 years, at 16.5%. While HRT use is increasing with a greater use of transdermal and vaginal formulations, the variation in the use of specific formulations also appears to be driven by socio‐economic factors. It is also possible that some of the country‐by‐country variations are driven by socio‐economic factors, with the highest rate of use in countries such as Finland [35]. A study in primary care settings from the United Kingdom by Hillman and colleagues [40] showed that the HRT prescribing rate in the most deprived quintile was 18% lower than in the least‐deprived quintile (adjusted incidence rate ratio [IRR] = 0.82; 95% CI = 0.77 to 0.86) after adjusting for relevant covariates. They [40] also reported that there was a significantly higher tendency to prescribe oral HRT than transdermal preparations (*P* < 0.001) in the general practitioner (GP) practices from more deprived areas. In summary, the HRT use decreased sharply after the early stoppage of the WHI trial and this decline in use continued until 2010, perhaps reducing the disparities in HRT use. The trend however has been reversed in recent years, and the use of HRT is increasing again, with a greater increase in the use of transdermal and vaginal preparations. The recent increase in use, however, appears to vary by socio‐economic factors and the women from the most deprived backgrounds have a higher likelihood of being prescribed oral HRT formulations [40]. Such variations by socio‐economic strata may increase health disparities.

## 3 Cancer burden in the EU/Europe attributable to HRT

The incidence of breast cancer varies widely within the EU [41], with age‐adjusted and standardised (ASR) incidence rates varying from 90/100000/year in Romania to more than twice that at 200/100 000/year in Belgium. As discussed above, the rates of HRT use also vary. Prior to the publication of WHI trial results [31, 32], only 1.4% of women aged 45–69 years in Italy used combined HRT as compared with 15% in Sweden [35]. Even though the variation reduced substantially [35] after the publication of WHI trial results, the HRT use is increasing since 2010 and differences in use by socio‐economic strata [40] mean that country‐by‐country variation will likely have increased. Furthermore, different HRT formulations have different magnitudes of increase in breast cancer risk and the risk is also modified by the duration of use. Together, these variations mean that any estimation of breast cancer burden attributable to HRT within Europe is fraught with substantial uncertainty. The population attributable fraction (PAF) varies from 0.2% for a short‐term (< 1 year) use of combined HRT by 2.0% of the population to 8.0% for a long‐term (> 5 years) use of combined HRT by 8.0% of the population. The number of cancers attributable to HRT at different prevalence of use of different formulations in the European Union is displayed in Table 1 (Table S1 for the same in 40 countries of Europe; Supplementary material—Annex S2). Country‐by‐country variation in use would mean that every year HRT could cause as few as four cancers in Bulgaria if 2% of women aged 45–69 years used combined HRT for less than 1‐year duration to 2533 cancers in metropolitan France if 8% of similarly aged women used combined HRT for more than 5 years.

**Table 1:** Population fraction and annual number of breast cancers attributable to hormone replacement therapy (HRT) at different prevalence of use in the European Union among women aged 45 and 69. HRT, hormone replacement therapy; PAF, population attributable fraction, calculated using the method originally described by Levin [42].

Formulation & duration	Relative risk	PAF/cancers attributable per year	Prevalence of HRT use (%)		
			2%	5%	8%
**Combined HRT; short‐term use**	1.11^a^, ^b^	PAF	0.22%	0.55%	0.87%
		Cancers attributable	412	1026	1636
**Combined dydrogestone‐based HRT; > 5 years use**	1.24^a^, ^b^	PAF	0.48%	1.19%	1.88%
		Cancers attributable	896	2224	3534
**Combined HRT (nondydrogestone‐based); > 5 years use**	2.08^c^	PAF	2.11%	5.12%	7.95%
		Cancers attributable	3966	9610	14 917
**Oestrogen‐only HRT; < 5 years use**	1.07^a^, ^b^	PAF	0.14%	0.35%	0.56%
		Cancers attributable	262	654	1045
**Oestrogen‐only HRT; > 5 years use**	1.15^a^, ^b^	PAF	0.30%	0.74%	1.19%
		Cancers attributable	561	1396	2224
	1.33^c^	PAF	0.66%	1.62%	2.57%
		Cancers attributable	1230	3045	4825

a Effect size from Vinogradova et al. [43];

b Effect size reported as Odds Ratio (OR), assumed to be equivalent to relative risk (RR) due to the low prevalence of the outcome of interest (< 10%) in the population studied and therefore OR not formally converted [44] to RR;

c Effect size from the Collaborative Group on Hormonal Factors in Breast Cancer [45].

It is also worth noting [41] that from 2010 onwards, breast cancer incidence plateaued or decreased in most countries in Europe, with the exception of 4 of the 6 Central and Eastern European countries (Bulgaria, Slovakia, Poland and Romania). Given the lag [34] in observing the impact of change in HRT use prevalence, some of this ecological variation in incidence may be attributable to declining HRT use from 2002 to 2010 as has been previously reported [46, 47, 48]. Although HRT use has been increasing since 2010, the type of formulations and doses [37] used mean that the impact may be smaller than before and the lag in effect may mean that it may not be observable with currently available data.

## 4 Recommendation for individuals

### 4.1 Scientific justification for update of the recommendation in ECAC5

The update recommendation on HRT in ECAC5 reads:

If you decide to use hormone replacement therapy for menopausal symptoms, after a thorough discussion with your healthcare professional, limit its use to the shortest duration possible.

The recommendation has been developed taking into account the new evidence (discussed below) since the publication of ECAC4 [27]. The main message of the current recommendation remains similar to that of the ECAC4: ‘Hormone replacement therapy (HRT) increases the risk of certain cancers. Limit use of HRT’ [27]. The main differences are that the updated recommendation underscores the importance of informed decision‐making and use of HRT only for menopausal symptom alleviation, that is, rules out use in asymptomatic individuals.

HRT has been classified as a carcinogen [1] as it is associated with an increased risk of developing breast, ovarian and endometrial cancers [27]. A large body of evidence was reviewed at the time of the ECAC4 recommendation [27]. However, substantial new evidence has become available since then and we discuss the implications of new evidence in the context of prior evidence.

#### 4.1.1 Evidence on the association between HRT and cancer

##### 4.1.1.1 HRT and breast cancer

The updated report of the WHI trial with more than 20 years of cumulative median follow‐up of 27 347 postmenopausal women [49] showed that conjugated equine oestrogen (CEE) in women with a prior hysterectomy was associated with lower (discussed below) breast cancer incidence [hazard ratio (HR), 0.78; 95% CI, 0.65–0.93; *P* = 0.005] and lower breast cancer mortality (HR, 0.60; 95% CI, 0.37–0.97; *P* = 0.04). The combined HRT [CEE with medroxyprogesterone acetate (MPA)] was associated with higher breast cancer incidence (HR, 1.28; 95% CI, 1.13–1.45; *P* < 0.001); breast cancer mortality was 35% higher, although this did not reach statistical significance (HR, 1.35; 95% CI, 0.94–1.95; *P* = 0.11).

**Fig. 2:**
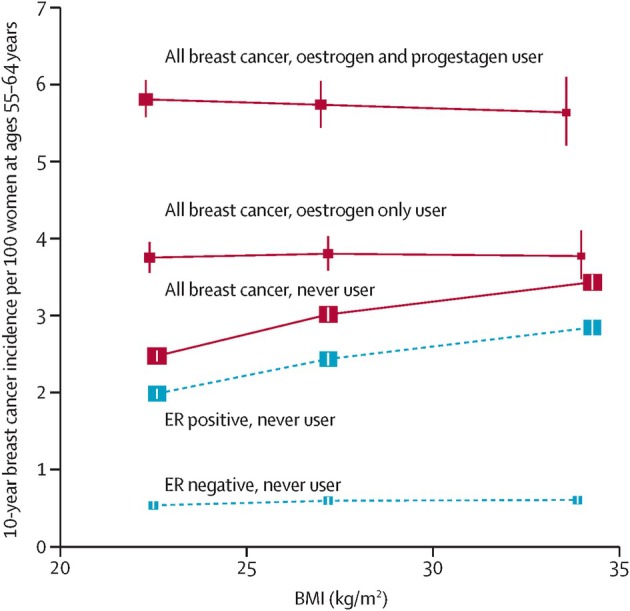
Relevance of BMI to the absolute 10‐year breast cancer incidence rate per 100 women at ages 55–64 years in never users and in current users of HRT. Adjusted relative risks for all breast cancers (red lines) during 5–14 years of current use were calculated taking never users with a BMI of 25–29 kg·m^−2^ as the reference and then standardising to the incidence rate of breast cancer in never users aged 55–64 years of average weight in western countries (i.e. 3 per 100 women). Separate results for ER^+^ and ER^–^ breast cancer are shown (both with broken blue lines) only for never users of HRT. BMI groups: < 25 kg·m^−2^ (lean); 25–30 kg·m^−2^ (overweight); and ≥ 30 kg·m^−2^ (obese); incidence is plotted against mean BMI values. BMI, body‐mass index; ER+, oestrogen‐receptor positive; ER–, oestrogen‐receptor negative; MHT, menopausal hormone therapy; HRT, hormone replacement therapy. Reproduced without modification from the report by the Collaborative Group on Hormonal Factors in Breast Cancer [45] under the terms of the Creative Commons CC‐BY licence.

The updated report from the Collaborative Group on Hormonal Factors in Breast Cancer [45] showed that every HRT type, except vaginal oestrogens, was associated with excess breast cancer risks, which increased steadily with the duration of use and these risks were greater for oestrogen–progestagen than oestrogen‐only preparations [45]. Among current users, these excess risks were observed even during Years 1–4 of use (oestrogen–progestagen RR 1.60, 95% CI, 1.52–1.69; oestrogen‐only RR 1.17, 1.10–1.26) and were twice as great during Years 5–14 (oestrogen–progestagen RR 2.08, 2.02–2.15; oestrogen‐only RR 1.33, 1.28–1.37) [45]. The risks did not differ by starting ages of 40–44, 45–49, 50–54 and 55–59 years but were attenuated in women starting HRT after 60 years of age. The risk was also attenuated with increasing adiposity, with no increase in risk for obese women using oestrogen‐only HRT. After cessation of HRT use, some excess risk persisted for more than 10 years; its magnitude depended on the duration of previous use, with little excess following less than 1 year of HRT use. In the context of prior evidence, the key findings from this updated report are that even short‐term use of HRT is associated with increased breast cancer risk and the excess risk may persist beyond 10 years depending on the duration of use. The reported interaction with adiposity is also noteworthy (Fig. 2).

The updated 20‐year breast cancer mortality data [50] from the Million Women study [51, 52] showed that current users as well as past users who used HRT for more than 5 years were at an increased risk of breast cancer death. The 20‐year breast cancer mortality rate ratios were 1.15 (95% CI, 1.01–1.32) and 1.35 (95% CI, 1.24–1.47) in current users of oestrogen‐only HRT with less than 5 years and more than 5 years of duration of use, respectively. The respective rate ratios in current users of combined HRT were 1.39 (95% CI, 1.27–1.53) and 1.64 (95% CI, 1.52–1.76). The past users who used HRT for more than 5 years (mean duration ~8 years) remained at 24% greater risk of breast cancer deaths [rate ratio, 1.24 (95% CI, 1.12–1.38)] as compared with non‐users [50].

Vinogradova et al. [43] conducted a large, nested case–control study in the UK general practices contributing to QResearch or Clinical Practice Research Datalink (CPRD). The study included 98 611 women aged 50–79 with a primary diagnosis of breast cancer matched to 457 498 female controls. The study provided contemporary effect size estimates in the UK general population for various HRT formulations. For example, among the combined oestrogen and progestogen formulations, the increased risk was highest for norethisterone (OR=1.88; 95% CI, 1.79–1.99) and lowest for dydrogesterone (OR=1.24; 95% CI, 1.03–1.48). Similar to the findings from the Collaborative Group on Hormonal Factors in Breast Cancer report [45], vaginal oestrogens were not associated with increased breast cancer risk and increasing adiposity attenuated the risk associated with oestrogen‐only HRT [43].

The evidence of association between oestrogen‐only HRT and breast cancer risk is not consistent across the study types. The observational studies [43, 45, 51] consistently report increased breast cancer risk with the use of oestrogen‐only HRT, whereas the WHI trial [49, 53] suggests protective effects of oestrogen‐only HRT. This discrepancy may be explained by time to HRT initiation from the menopause [54] and the interaction between oestrogen‐only HRT and adiposity [45, 54]. Endogenous sex hormones including oestradiol are associated with increased risk of postmenopausal breast cancer [55, 56]. Data also suggest that the increase in breast cancer risk with increasing BMI among postmenopausal women is largely the result of the associated increase in oestrogens, particularly bioavailable oestradiol [57]. However, there may be a ‘ceiling effect’ and further increase in oestradiol levels through exogenous use of oestrogen‐only HRT may not further increase the breast cancer risk. Second, CEE is pharmacologically distinct from 17β‐estradiol‐based HRT. CEE contains a complex mixture of oestrogens, such as estrone sulfate, and other conjugates, which may interact differently with the breast tissue and oestrogen receptors compared to conventional HRT. These differences in pharmacology may also account for the reduced risk observed in the WHI trial. The updated results from the Collaborative Group on Hormonal Factors in Breast Cancer report [45] do not show an increase in breast cancer risk for obese women using oestrogen‐only HRT and the results of the case–control study by Vinogradova and colleagues also show that increasing adiposity attenuated the risk associated with oestrogen‐only HRT [43]. In the oestrogen‐alone component of the WHI trial [49], almost 80% of the participants were overweight or obese and almost half of the participants were obese. Therefore, the lack of increase in breast cancer risk with CEE in the WHI trial [49] is not inconsistent with recent evidence once the interaction with adiposity and pharmacology is taken into account (Fig. 2).

It is worth noting that the magnitude of excess breast cancer risk varies not only by duration of HRT use but also by the formulation used. The evidence consistently points to vaginal oestrogen‐only HRT as not being associated with an increased risk of breast cancer [43, 45, 58, 59]. Among the combined HRT formulations, those containing dydrogestone [43] and micronised progesterone [60] are associated with the smallest increase in breast cancer risk; the latter may be associated with a greater increase in endometrial cancer risk [61]. The importance of considering the specific formulation of HRT when interpreting breast cancer risk and making clinical decisions cannot be overemphasised.

To summarise the association between HRT use and breast cancer, the use of any HRT except for vaginal oestrogen, even for a short duration, is associated with increased breast cancer risk. Except for short durations (< 1 year) of use, the excess risk persists beyond 10 years after cessation of use, and the magnitude of residual excess risk is proportional to the duration of use. The magnitude of increase in risk is the smallest for oestrogen‐only HRT, which can only be used in women who have undergone hysterectomy. In obese postmenopausal women, who are at an increased risk of developing breast cancer, oestrogen‐only HRT does not increase their risk further. Among the combined HRT formulations, dydrogesterone‐containing HRT is associated with the smallest increase in breast cancer risk.

##### 4.1.1.2 HRT and ovarian cancer

Multiple meta‐analyses [62, 63, 64] of observational studies report an association between HRT use and increased risk of ovarian cancer; pooled RR 1.29 (95%CI, 1.19–1.40; *I* ^2^ = 57.4%) [62]. Early evidence suggested the risk to be associated with oestrogen‐only HRT, but recent studies suggest a similar risk association with combined HRT as well [63]. The association however differs by histological subtype of ovarian cancer. HRT use is associated with an increased risk of serous ovarian cancer (RR, 1.50; 95% CI, 1.35–1.68) [62], and it may be [62, 63] associated with increased risk of endometrioid ovarian cancer. HRT does not appear to increase the risk of clear cell, or mucinous ovarian cancer [62].

##### 4.1.1.3 HRT and endometrial cancer

A systematic review of 28 studies [61] suggests that all HRT formulations, with a possible exception of continuous combined HRT, increase the risk of endometrial cancer, even when treatment lasts less than 5 years. The increase in risk appears to be greater with the use of micronised progesterone.

##### 4.1.1.4 HRT and colorectal cancer (CRC)

HRT may reduce the risk of colorectal cancer [65]; however, the results from randomised trials are inconsistent. The HERS trial [66] did not observe a protective effect (HR, 0.81; 95% CI, 0.46–1.45), whereas the WHI trial [31] suggested a protective effect of HRT (HR, 0.63; 95% CI, 0.43–0.92). A systematic review [67] of four randomised controlled trials, eight cohort studies and eight case–control studies found that combined HRT (RR, 0.74; 95% CI, 0.68–0.81), and oestrogen‐only HRT (RR, 0.79; 95% CI, 0.69–0.91) decreased the CRC risk. The effect of HRT may be subtype‐specific. A systematic review [68] of observational studies reported that HRT reduced the risk of microsatellite stable CRC (RR, 0.80; 95% CI, 0.73–0.89) but not that of CRC with high microsatellite instability (MSI‐H CRC) (RR, 1.02; 95% CI, 0.85–1.21).

#### 4.1.2 Presentation of the recommendation

##### 4.1.2.1 Equity

The substantial decrease in the rate of HRT use between 2002 and 2010 reduced inequities [35]. However, with the HRT use on the rise again, and with the socio‐economic variation discussed above, the greater HRT use will increase worsen health inequity. This individual recommendation stresses the importance of informed choice and using HRT for as short a duration as possible. The policy recommendation (Table 2) outlines the framework for creating an enabling environment to make such an informed choice. Appropriate implementation and adherence to the recommendation will reduce inequities.

**Table 2:** European Code Against Cancer, 5th edition: recommendation for policymakers on hormone replacement therapy (HRT).

Hormone replacement therapy
**Make provisions for:** ○ Easy access to healthcare professionals for women to discuss their menopausal symptoms and the benefits and harms of using hormone replacement therapy (HRT) and nonhormonal alternatives. ○ Assessment of baseline cancer risk, including mammography before starting to use HRT, where applicable. ○ Availability, on a prescription‐only basis, of various formulations to personalise use of HRT and minimise risks. ○ Periodic re‐evaluation of symptoms and HRT use.

© 2025 International Agency for Research on Cancer / WHO. Used with permission. References:• Menopause: Identification and Management. National Institute for Health and Care Excellence (NICE) guideline NG23. London: NICE; 2024. Available from: https://www.nice.org.uk/guidance/ng23 [69]. [Correction added on 08 January 2026: The credit line was added.]

##### 4.1.2.2 Suitability, actionability and acceptability of the recommendations for the individual

The recommendation takes into account the nuances of menopausal symptom management without being rigidly prescriptive. It underscores the importance of individual autonomy and informed choice; it is therefore suitable, actionable and likely acceptable across the populations in the EU.

### 4.2 Cobenefits for prevention of non‐communicable diseases other than cancer with similar risk factors and opportunities for health promotion

Attaining menopause increases a woman's risk of fractures due to a decline in bone mineral density and coincides with an increase in the risk of cardiovascular disease, diabetes as well as cognitive decline [70]. HRT was therefore anticipated to ameliorate these risks and the trials [31, 66, 71] were designed to test this hypothesis. However, contrary to expectations, HRT was associated with an increased risk of coronary heart disease (CHD), stroke [31] and dementia [71]. The most recent update [65] of evidence for the US Preventive Services Task Force (USPSTF) based on 20 trials (*N* = 39 145) and three cohort studies (*N* = 1 155 410) shows that HRT (both oestrogen‐alone and combined) reduced the risk of fractures and diabetes but significantly increased the risk of gallbladder disease, stroke, venous thromboembolism and urinary incontinence.

A systematic review [72] of 23 studies reported that HRT use had a significant negative effect on global cognition, and this effect might be especially more visible for those aged more than 60 years and with more than 6 months of use. However, a systematic review [73] of 34 randomised trials reported time‐dependent effects of HRT on certain aspects of cognition, with variations based on formulation and timing of initiation. For example, the duration of treatment >1 year was associated with worsening in visual memory as compared to shorter duration. A recent nested case–control study in UK general practices contributing to Qresearch or CPRD reported mixed findings [74]. The global risk of dementia was lower in women younger than 80 years who had been taking oestrogen‐only therapy for 10 years or more (OR, 0.85; 95% CI, 0.76 to 0.94), but the risk of Alzheimer's disease was higher in women who had used combined HRT for 5–9 years (OR, 1.11; 95% CI, 1.04 to 1.20) and for 10 or more years (OR, 1.19; 95% CI, 1.06 to 1.33) [74]. A recent Danish national nested case–control study [75] reported increased risk of all‐cause dementia with combined HRT (HR, 1.24; 95% CI, 1.17 to 1.33) and a duration‐response relationship. Given the mixed nature of evidence, it is unlikely that the benefits (if any) of HRT on dementia risk will outweigh the harms.

The current evidence points to a lack of net benefit and a possible risk of harm with the use of HRT for the prevention of non‐communicable diseases other than cancer in asymptomatic individuals. The USPSTF recommends against the use of HRT for the primary prevention of chronic conditions in postmenopausal persons [76]. The ECAC5 recommendation similarly refers to considering HRT use only for menopausal symptoms, excluding any use in asymptomatic individuals.

### 4.3 Other drugs/medical therapies and cancer

The ECAC5 Working Group on Medical interventions also considered medical therapies/interventions that have associations with cancer risk and are applicable to the general population. We did not consider drugs/therapies such as antineoplastic agents, SERMs, AIs and 5‐ARIs since these are used in specific subpopulations not in the general population. Hormonal contraceptives and aspirin on the other hand are medical therapies that are used in the general population and were therefore evaluated to ascertain if a relevant recommendation should be made. After careful consideration, the Working Group decided against making a specific recommendation regarding these common medical therapies; the relevant evidence and the scientific rationale underpinning this decision are discussed below.

#### 4.3.1 Hormonal contraceptives

Hormonal contraceptives (HC) are classed as carcinogens [1] due to the increased risk of breast cancer [77, 78, 79, 80], cervical cancer [81] and possibly liver cancer [82]. However, HCs also reduce the risk of ovarian cancer [83], colorectal [84] and endometrial cancer [85, 86, 87]. HCs are used at a younger age, at which baseline cancer risk is low, and as a result, the absolute excess risk of cancer is very small. Fitzpatrick et al. [77] reported that the 15‐year absolute excess breast cancer risk associated with 5 years of use of oral combined or progestogen‐only contraceptives in high‐income countries was estimated at: 8 per 100 000 users from age 16 to 20 years and 265 per 100 000 users from age 35 to 39 years. Turati et al. [88] estimated population attributable and prevented fractions combining relative risks and prevalence of exposure in Italian women. Applying HC effects on breast, cervical, colorectal, ovarian and endometrial cancers, they estimate that oral HC use prevented 1174 cancer diagnoses and 577 cancer deaths. HCs also have other sexual and reproductive health implications, and the Working Group therefore elected not to recommend against HCs even though these are classed as carcinogens.

#### 4.3.2 Aspirin

Aspirin has been shown to prevent the development of certain cancers as well as deaths due to certain cancers [7, 89, 90, 91, 92, 93, 94, 95]. Various mechanisms of action [6, 94] have been postulated including prevention of metastasis mediated through inhibition of Thromboxane A_2_ leading to the reversal of suppression of T‐cell immunity [6, 96]. It is important to carefully consider the peculiarities of aspirin's action when assessing literature evidence. (a) All the current evidence suggests that aspirin's effects on cancer are site‐specific and not tumour‐agnostic, with the largest effects seen on colorectal cancer [93, 94]. (b) It takes at least 3 and 5 years, respectively, for aspirin's effect on cancer incidence and cancer deaths to become apparent [90], indeed its effect in the Women's Health Study (WHS) only became apparent in the post‐treatment period after 10 years of follow‐up [93]. Therefore, any results of an aspirin trial with a follow‐up of less than 10 years should be interpreted with caution [97] and a long‐term follow‐up of all aspirin trials is necessary. (c) Aspirin needs to be used for a minimum duration of 5 years before its anticancer effects are observed. (d) Current evidence also indicates that the anticancer effects are observed at low doses (30‐40 mg per day) of aspirin when antiplatelet action is the main mechanism [6, 94]. The anti‐inflammatory action of aspirin requires daily administration of 2 g of aspirin in multiple divided doses, a dose no longer used in clinical practice. Although several other mechanisms [6, 94] that may come into action at doses higher than the antiplatelet dose have been proposed, these are yet to be validated in clinical studies. As the main anticancer effect occurs at low doses, the dose–response test often used in epidemiological assessment does not apply to aspirin; instead, it is important to assess the duration–response relationship.

In 2016, the USPSTF [8] recommended aspirin for prevention of cardiovascular disease and colorectal cancer. However, in the 2022 update [98] of its recommendation, the USTSPF removed the CRC indication for use of aspirin as a primary prevention. The updated evidence synthesis [99] and a modelling study [100] for the 2022 recommendation included results from relatively short‐term follow up of ASPREE [101], ARRIVE [102] and ASCEND [103] trials. The ARRIVE [102] and ASCEND [103] trials did not show any anticancer beneficial effects of aspirin, whereas the ASPREE [101] trial observed excess all‐cause and cancer mortality in the aspirin arm. Apart from the short follow up, several other issues (e.g. heterogeneity within trial, selection bias and possible cause‐of‐death misattribution bias) in the ASPREE trial merit consideration in order to assess evidence in correct context but a full discussion of these is outside the scope of this paper. Inclusion of the data from these trials increased uncertainty regarding anticancer effect and reduced effect size in the evidence synthesis [99] and modelling study [100] leading to removal of CRC indication.

Following the IARC methodology to include a new recommendation in ECAC5 [28], the ECAC5 Working Group on Medical interventions commissioned an umbrella review to address the question: ‘What is the balance of benefits and harms of using Aspirin for cancer prevention in the general population?’

The overview included six systematic reviews regarding the balance of benefits and harms of using aspirin (75–350 mg·day^−1^) for 5 years or more for preventing cancer published in English over the 5 years until July 2023. Two of these systematic reviews synthesised evidence from randomised trials and both were graded as low quality as per AMSTAR‐2 criteria; four systematic reviews synthesised evidence from observational studies and 1 was graded as low quality while three were graded as very low quality as per AMSTAR‐2 criteria. The use of aspirin for 5 years or more in the general population, when compared to not using aspirin, may have a trivial effect on overall cancer incidence at 5 to 10 years (RR, 1.01; 95% CI, 0.90–1.14) and 10 or more years (RR, 1.00; 95% CI, 0.94–1.06) (low certainty). Aspirin may decrease the incidence of CRC (RR, 0.90; 95% CI, 0.78–1.04) and prostate cancer (RR, 0.82; 95% CI, 0.80–0.95) (low certainty). Aspirin's effect on oesophageal, lung, breast and gastric cancer is uncertain (very low certainty).

Aspirin may reduce cancer mortality within 5–10 years (RR, 0.92; 95% CI, 0.84–1.01); however, the effect is absent after 10 years (RR, 1.00; 95% CI, 0.88–1.14) (low certainty). The reduction in the risk of CRC mortality (RR, 0.80; 95% CI, 0.51–1.27) has not been observed (very low certainty). Aspirin probably increases the risk of major bleeding, (RR, 1.34; 95% CI, 1.17–1.53) (moderate certainty).

After careful consideration of the evidence from the umbrella review, low certainty of evidence and excess cancer mortality in the ASPREE trial, the Working Group therefore elected not to recommend aspirin for cancer prevention in the general population. However, it is worth noting that the remit of this ECAC5 Working Group is assessment in the average‐risk general population. Assessment of the benefit‐harm balance in specific population subgroups, for example, those at high risk of CRC is outside the current remit. Such assessment may find the benefit‐harm balance in favour of recommending aspirin as a primary prevention agent, and it would not be discordant with the lack of recommendation by the ECAC5 Working Group.

## 5 Recommendation for policymakers

### 5.1 Presentation of the recommendations for policymakers and key stakeholders

The principle underpinning the policy recommendations (Table 2) is that although HRT is an established carcinogen, the use of HRT might be beneficial for some women in the treatment of menopausal symptoms, particularly when nonhormonal treatment options do not provide sufficient clinical effect. The policy framework should therefore ensure that healthcare systems can minimise individuals' exposure to HRT as much as possible in these circumstances to mitigate risks associated with HRT use. To that end, an individual must be empowered to make an informed decision regarding HRT use. The healthcare systems should be able to ensure that the HRT formulation(s) being prescribed are tailored to individual needs with as low a dose as possible and arrangements for periodic evaluation are in place to limit exposure to HRT.

#### 5.1.1 Key policies

**Fig. 3:**
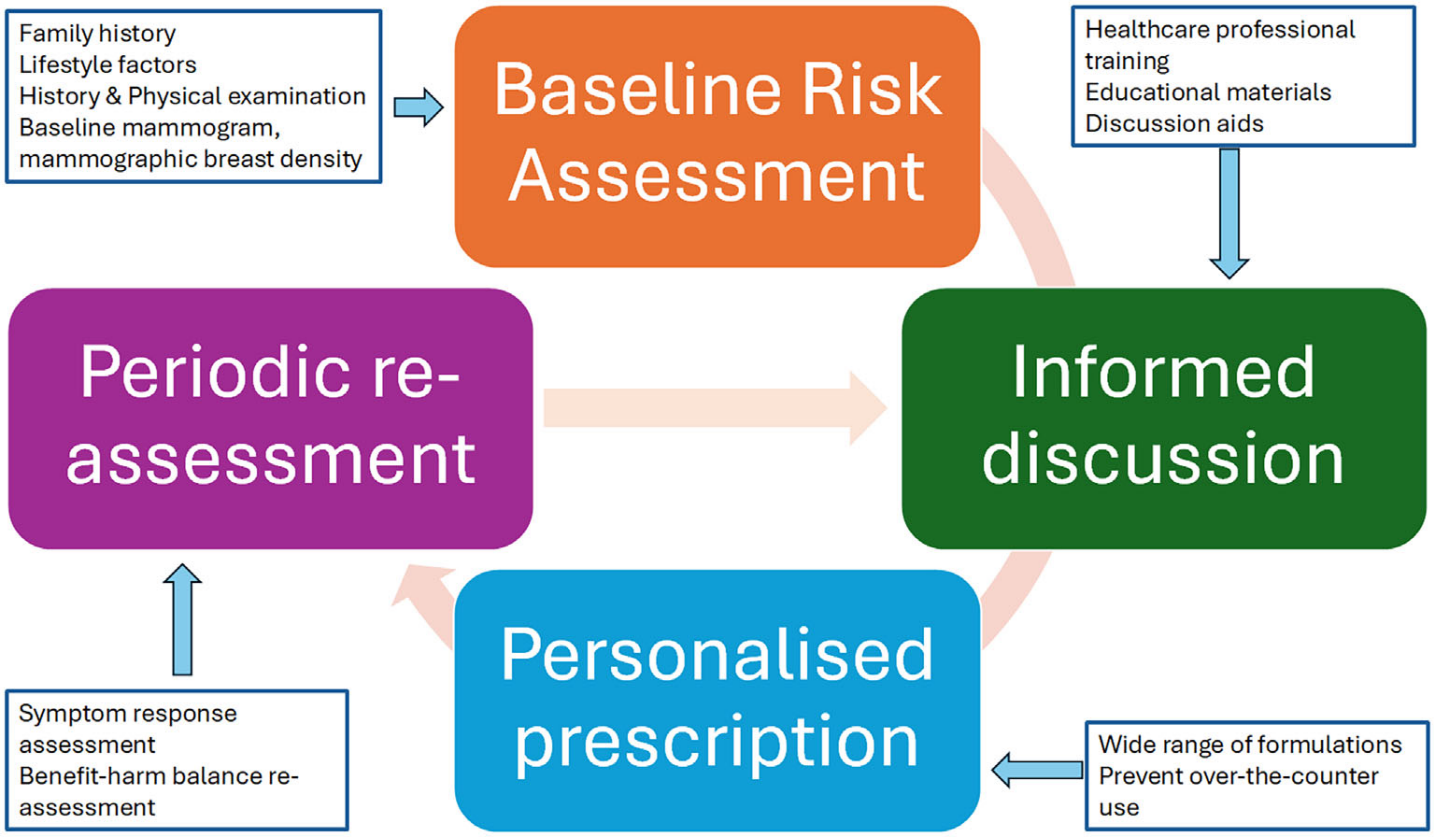
Key policy considerations for creating an enabling environment. Figure developed by authors based on evaluation of existing evidence and guidelines from scholarly societies or organisations [69, 104, 105]. It highlights the key considerations for policymakers in order to create enabling environment for the safest possible use of HRT by individuals.

The UK National Institute for Health and Care Excellence (NICE) updated its guidance on the identification and management of menopause in November 2024 [69]. Apart from the review of evidence and clinical guidance, the document also provides guidance as well as resources for the implementation of the guidance (Tools and Resources). Creating an enabling environment is necessary so that individuals can make an informed decision and successfully adhere to the recommendations. To that end, the key considerations for policymakers and stakeholders are (a) baseline risk assessment, (b) informed discussion with healthcare professionals, (c) availability of a wide range of formulations on a prescription‐only basis and (d) provision for periodic assessment (Fig. 3).

#### 5.1.2 Creating enabling environments for informed decision making

Informed shared decision‐making regarding HRT use needs discussion of the balance between benefits and harms. Any HRT‐associated absolute excess risk of breast cancer for an individual would depend on their baseline risk. Therefore, a thorough assessment of baseline breast cancer risk in an individual is an essential component of service provision. Mammographic breast density (MBD) is a risk factor for the development of breast cancer [106]. HRT increases MBD and reduces the sensitivity of mammographic screening [107]. Growth of pre‐existing breast cancer may also be fuelled by HRT. For these reasons, a baseline mammogram prior to initiation of HRT might be a part of baseline risk assessment. Healthcare professionals need training, continued professional development and access to educational material [104, 105] and tools such as discussion aids [69] so that baseline risk assessment as well as the discussion of balance of benefits and harms can be appropriately implemented.

#### 5.1.3 Feasibility and resources required

The updated NICE guidance [69] outlines measures to provide ‘access to healthcare professionals with expertise in menopause’, provides discussion aid tools and lists useful educational resources. Management of menopausal symptoms should first be attempted using non‐hormonal approaches [108]. Newer agents such as selective neurokinin 3 receptor antagonist fezolinetant [109, 110] and neurokinin‐1,3 (dual) receptor antagonist elinzanetant [111] may soon become widely available for the management of vasomotor symptoms. Cognitive behavioural therapy (CBT) is one such established approach for the management of vasomotor symptoms [112, 113]. The NICE guidance [69] also provides resources for ‘access to cognitive behavioural therapy (CBT)’. National/regional healthcare systems should similarly make provisions for training and continued professional development of healthcare professionals with expertise in menopause. To prevent misuse by asymptomatic individuals, HRT should be available on a prescription‐only basis. A legislative/administrative framework would also be necessary to prevent over‐the‐counter sales or online sales of HRT without prescription. HRT use needs to be tailored to individual needs; healthcare professionals should have a wide range of formulations available to prescribe based on the guidance provided by specialty organisations [104, 105]. The importance of periodic reassessment of symptoms and therapy cannot be overemphasised [104, 105] in order to ensure that HRT is used for as short a duration as possible. It is necessary to be compliant with the broader principle that any prescription‐only medication should be initiated or discontinued only under medical supervision. Policymakers and healthcare systems will need to assess resource impact and make workforce as well as pharmacy provisions accordingly.

## 6 Conclusions

Principles of the 5th revision of the European Code against Cancer were that the recommendations are (a) based on scientific evidence of carcinogenicity or modification of cancer risk and are relevant to the EU, (b) suitable, actionable and acceptable at the individual level, (c) the recommendation can be clearly communicated to the general population and (d) existing international policies support the recommendation for the individual [28]. After careful consideration of available evidence for HRT, hormonal contraceptives and aspirin, the Working Group elected against making any specific recommendation for hormonal contraceptives as the overall benefit‐harm balance is in favour of using these. The working group also elected against making any specific recommendation for aspirin due to low certainty of benefit in the general population. Despite the carcinogenic potential of HRT, its use may be appropriate for a limited period to alleviate symptoms associated with menopause. Any such use should be preceded by a baseline assessment of breast cancer risk and a thorough discussion of the benefit‐harm balance with a healthcare professional, so the women have the opportunity of making an informed choice. HRT should be used for as short a duration as possible. Use of HRT by asymptomatic individuals for the prevention of chronic conditions is strongly discouraged. To empower individuals to make an informed decision regarding HRT, easy access to healthcare professionals with expertise in menopause and breast cancer risk assessment, availability of a wide range of formulations on a prescription‐only basis and periodic monitoring of symptoms and HRT use are required. Policymakers and healthcare systems should develop strategies to facilitate this.

## Conflict of interest

All authors declare no competing interests. Where authors are identified as personnel of the International Agency for Research on Cancer/World Health Organization, the authors alone are responsible for the views expressed in this article and they do not necessarily represent the decisions, policies, or views of the International Agency for Research on Cancer /World Health Organization.

## Author contributions

MAT was responsible for writing the first version of the manuscript. All authors gave critical revisions on the intellectual content of the manuscript and approved the final manuscript.

## Supporting information


**Annex S1.** European Code Against Cancer, 5th edition. © 2025 International Agency for Research on Cancer / WHO. Used with permission. [Correction added on 08 January 2026 after first online publication: The credit line was updated.]
**Annex S2**. Population fraction and annual number of breast cancers attributable to HRT at different prevalence of use in Europe (40 countries) among women aged 45 to 69.

## Data Availability

The data that support the findings of this study are available in Table 1 and the supplementary material of this article.
